# Automated Detection and Screening of Traumatic Brain Injury (TBI) Using Computed Tomography Images: A Comprehensive Review and Future Perspectives

**DOI:** 10.3390/ijerph18126499

**Published:** 2021-06-16

**Authors:** Vidhya V., Anjan Gudigar, U. Raghavendra, Ajay Hegde, Girish R. Menon, Filippo Molinari, Edward J. Ciaccio, U. Rajendra Acharya

**Affiliations:** 1 Department of Computer Science and Engineering, Manipal Institute of Technology, Manipal Academy of Higher Education, Manipal 576104, India; vidya.prakash@manipal.edu; 2 Department of Instrumentation and Control Engineering, Manipal Institute of Technology, Manipal Academy of Higher Education, Manipal 576104, India; anjan.gudigar@manipal.edu; 3 Institute of Neurological Sciences, Glasgow G51 4LB, UK; dr.ajayhegde@gmail.com; 4 Department of Neurosurgery, Kasturba Medical College, Manipal Academy of Higher Education, Manipal 576104, India; girish.menon@manipal.edu; 5 Department of Electronics, Politecnico di Torino, 24 Corso Duca degli Abruzzi, 10129 Torino, Italy; filippo.molinari@polito.it; 6 Department of Medicine, Columbia University, New York, NY 10032, USA; ciaccio@columbia.edu; 7 School of Engineering, Ngee Ann Polytechnic, 535 Clementi Road, Singapore 599489, Singapore; aru@np.edu.sg; 8 Department of Biomedical Engineering, School of Science and Technology, SUSS University, 463 Clementi Road, Singapore 599491, Singapore; 9 Department of Bioinformatics and Medical Engineering, Asia University, Taichung 41354, Taiwan

**Keywords:** traumatic brain injury (TBI), CAD, computed tomography, intracranial hematoma, elevated ICP, midline shift

## Abstract

Traumatic brain injury (TBI) occurs due to the disruption in the normal functioning of the brain by sudden external forces. The primary and secondary injuries due to TBI include intracranial hematoma (ICH), raised intracranial pressure (ICP), and midline shift (MLS), which can result in significant lifetime disabilities and death. Hence, early diagnosis of TBI is crucial to improve patient outcome. Computed tomography (CT) is the preferred modality of choice to assess the severity of TBI. However, manual visualization and inspection of hematoma and its complications from CT scans is a highly operator-dependent and time-consuming task, which can lead to an inappropriate or delayed prognosis. The development of computer aided diagnosis (CAD) systems could be helpful for accurate, early management of TBI. In this paper, a systematic review of prevailing CAD systems for the detection of hematoma, raised ICP, and MLS in non-contrast axial CT brain images is presented. We also suggest future research to enhance the performance of CAD for early and accurate TBI diagnosis.

## 1. Introduction

Traumatic brain injury (TBI) arises when sudden and direct/indirect external forces, such as a bump, blow to the head, or other kind of injury, result in neuropathological damage and brain dysfunction. TBI can result in significant disruption in the normal functioning of the brain, leading to temporary or permanent neurological deficits. This silent epidemic [1] affects millions of people worldwide annually, with high morbidity and mortality rates. It is estimated that 1.7 million people suffer TBI every year [2] in the United States, with total lifetime TBI medical expenses that are expected to be approximately $76.5 billion [3]. According to the Indian Head Injury Foundation (IHIF), India has the highest rate of brain injury in the world; one out of six TBI patients die, and most cases of death ensue within two hours after the injury [4].

The brain damage due to TBI results in a heterogeneous group of injuries that can distort normal brain function, resulting in cognitive, physical, emotional, and behavioral disability [5]. The complications occur directly or indirectly after the trauma, and hence, the injuries following TBI can be predominantly categorized as primary and secondary injuries [6]. Primary injuries are the result of the direct impact of trauma that includes extradural, subdural, and intracranial hemorrhage (ICH) and diffuse axonal injury (DAI) [5,7]. The abrupt external mechanical forces can rupture the blood vessels, and the blood starts accumulating in various intracranial compartments of the brain, leading to hemorrhage. The hematoma can be categorized as intra-axial hematoma and extra-axial hematoma, respectively, based on its occurrence with respect to the brain substance. Intra-axial hematoma includes intracerebral hemorrhage (ICH), subdural hematoma (SDH), sub-arachnoid hematoma (SAH), and intraventricular hematoma (IVH), whereas extra-axial hematoma consists of epidural hematoma (EDH) [8]. The mortality rate of ICH is nearly 50% within the first year [9]. The primary injury can appear within a short period of 100 milliseconds [5], and the health status of the patient starts declining within the first few hours after its onset.

**Figure 1:**
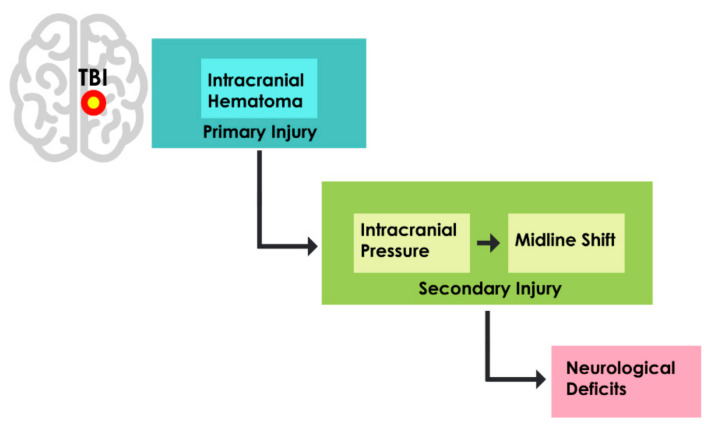
Relationship between hematoma and secondary injuries in TBI.

Secondary injuries start developing from minutes to days after the primary brain insult, which comprises a series of molecular, chemical, inflammatory, and metabolic alterations [6]. Secondary injuries include elevated or raised intracranial pressure (ICP), midline shift (MLS), herniation, ischemia, infarction, hydrocephalus, cerebral vasospasm, etc. [5,8]. Some of the devastating and lethal consequences of intracranial hematoma are a raised or elevated intracranial pressure and midline shift [10,11], as depicted in Figure 1.

The adult cranium is a stiff box of constant volume consisting of blood, brain, and cerebrospinal fluid (CSF). The Monro–Kellie doctrine states that the sum of the volumes of these three major components remains constant [12]. Therefore, in conjunction with the increase in volume of any of the intracranial contents, the volume of at least one of the two components should be reduced. Furthermore, this potential increase in volume will in turn lead to elevated ICP levels. The displacement of CSF and blood into the intracranial space will slowly occur owing to the expansion of the hematoma inside the rigid cranium. During the initial phase of hematoma growth, the ICP levels remain low due to effective management per the Monro–Kellie doctrine. When the progressive expansion of hematoma approaches a certain limit, the compensatory mechanisms will get exhausted, and further displacement of CSF or blood is not possible. Hence, the entire equilibrium is disrupted, leading to raised ICP [11]. Exacerbated ICP levels have proven to result in a worse outcome [10,13], and can damage various brain structures, leading to midline shift, brain herniation, and even death [10,14].

The unmitigated ICP levels due to the mass effect of hematoma can displace the midline anatomical structures to the sides of the brain, leading to the condition termed midline shift (MLS). Due to the symmetry of the brain structure, the midline can be considered as an imaginary central line, which is straight in normal, healthy subjects [15]. The displacement of any of the three brain midline structures, namely the septum pellucidum (SP), third ventricle (V3), or pineal gland (PG), from the ideal midline, is considered for the computation of the degree of MLS. The mass effect of hematoma generates high intracranial pressure, thereby shifting the brain from its central position, and results in the compression of brain structures. This can eventually lead to death. Therefore, MLS is considered a significant indicator of ICP, and a strong predictor of worst patient outcomes after TBI. The degree of shifting of SP with respect to the ideal midline is widely used to quantify MLS, and a shift in the midline greater than 5 mm [16] necessitates immediate surgery to invasively remove the acute hematoma.

Non-contrast computed tomography (CT) is the preferred modality of choice for the diagnosis and management of TBI in the acute setting, as it is fast, widely available, and offers good contrast between blood and brain tissues [7,17]. Detection of hematoma in the CT scans and assessment of three major determinants, namely location, volume, and size [7,18], is crucial for prognosis and decision-making. The gold standard for monitoring ICP involves the use of an external ventricular drain (EVD), an invasive procedure that is highly susceptible to infections and complications [19,20]. Furthermore, the lack of invasive ICP monitoring and trained neurosurgeons in various clinical settings necessitates the need of CT scans to detect raised ICP [14]. Multiple signs in CT images, such as effacement of basilar cisterns, midline shift, and hematoma volume, can be used to predict ICP [7,14,21]. Manual inspection and quantification is the current clinical practice to quantify MLS.

The patient outcomes after TBI can be greatly enhanced by the rapid and accurate extraction and management of the information present in the CT images. However, lack of reliable and efficient automated tools to analyse and interpret have limited the maximum utilisation of details present in CT scans for prompt and early management of TBI. Various research studies show that the proper visual inspection and manual estimation of TBI outcome based on CT are time-consuming, subject to inter-observer and intra-observer variabilities, and prone to inadvertent error and misdiagnosis [2,22,23]. Quick selection and appropriate interpretation of CT slices requires high expertise, which may not be possible for junior radiologists or emergency care physicians, especially in the case of review at odd hours. The initial interpretation by inexperienced readers is often tedious, and results in misinterpretation and clinical consequences [24,25]. Moreover, manual segmentation of hematoma or midline structures from selected CT slices is challenging due to reasons such as variation in pixel-wise intensity, uneven boundaries, high contrast of tissues, and the presence of noise and artefact [26,27,28]. Furthermore, the set of features required for CT-based ICP estimation cannot be readily identified by visual inspection, and is also subject to intra-observer and inter-observer variability [29]. Moreover, the measurement of MLS should be carried out at the level of the foramen of Monro based on clinical guidelines, and hence, the selection of the appropriate CT slice is crucial [30]. MLS in smaller amounts is difficult to detect from CT imagery. As the amount of shift is vital to assess the extent of brain damage, the precision of quantification is important for decision-making and further diagnosis. Therefore, the use of computer aided diagnosis (CAD) systems can bring significant reduction in human error and provide quantitative and qualitative assessments of TBI rapidly and accurately, thereby leading to improved clinical outcome.

The main aim of a typical CAD system is to decrease the false negative rates by identifying the features that are normally used by the clinicians to detect the abnormality [31]. The ever-growing research initiatives have extended the CAD systems to perform various image analysing techniques that enable clinicians to detect disease, plan treatment, predict risk, and determine prognosis. The interpretation provided by the CAD systems can be utilised by radiologists as a supplementary tool for final decision-making. CAD systems equipped with machine learning and deep learning techniques can quickly learn and predict the abnormalities present in larger datasets. The CAD-assisted detection systems are usually composed of various image processing techniques to perform pre-processing, segmentation, feature extraction, feature selection, and classification. Several CAD-based approaches are proposed to diagnose brain abnormality in imagery, as represented using different modalities [32,33,34]. These semi-automated or fully automated approaches are applied to detect either a single brain abnormality or multiple pathologies in a supervised or unsupervised fashion [33,34], and deploy machine learning or deep learning techniques to enhance accuracy and efficiency [32]. A general categorisation of various approaches employed by CAD systems to assess TBI is shown in Table 1.

## 2. Search Strategy and Organisation of the Review

**Figure 2:**
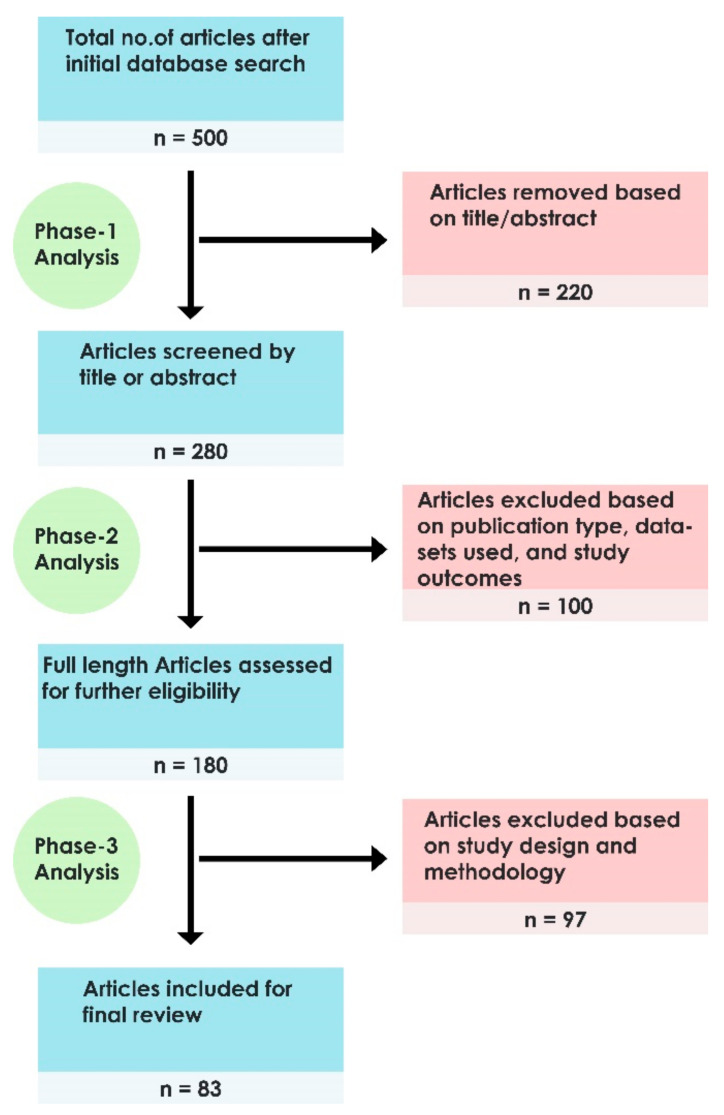
Flow diagram of the article selection process.

The relevant research literature for the study was obtained by conducting searches in PubMed, Scopus, Web of Science, IEEEXplore, ScienceDirect, and Google Scholar databases. Figure 2 depicts the article selection process used in the study.

A three-phase analysis was conducted to select suitable articles from the initial search results. In the first phase of analysis, 500 published articles were assessed based on the title and abstract, and 280 publications were identified. The articles were re-assessed in the second phase of analysis with respect to the publication type, datasets used, and outcomes of the research study. Following the second phase of analysis, 180 articles remained. The eligible articles for final review were identified in the third phase of analysis, which involved the examination of the study design and methodology. A total of 83 articles were shortlisted and included in the study.

The set of keywords used, individually or combined, included ‘traumatic brain injury’, ‘automated intracranial hematoma’, ‘automated intracranial haemorrhage’, ‘hematoma’, ‘segmentation’, ‘automated ICP prediction’, ‘MLS estimation’, ‘computer-aided diagnosis’, ‘intracranial pressure levels’, ‘brain midline shift’, ‘automatic detection and classification’, ‘CT images’, and ‘quantification’. The inclusion and exclusion criteria used in the article selection process are outlined in Table 2.

**Figure 3:**
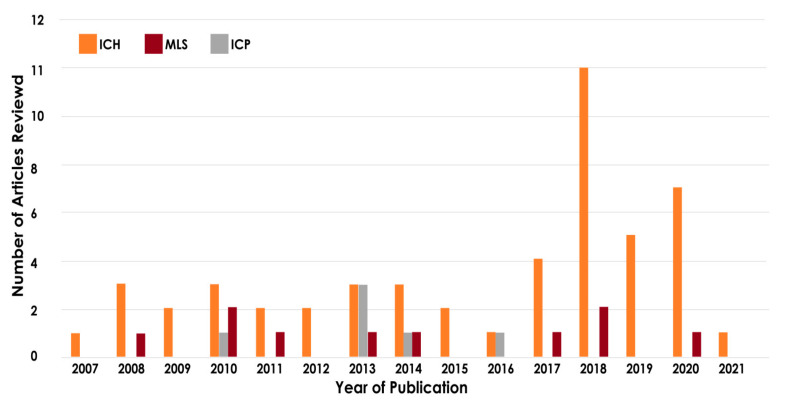
Year-wise distribution of papers reviewed for assessing TBI based on ICH, ICP, and MLS.

The year-wise distribution of the papers reviewed is shown in Figure 3. It can be inferred that the research work related to MLS and ICP using CT images for assessing TBI is still in its infancy, and hence, there is significant room for improvement.

The remainder of this review is organised as follows. In Section 3, the different publicly available datasets to develop high-performance CAD systems are discussed. In Section 4, the various approaches to develop CAD systems for TBI assessment are provided, with a highlight of the characteristics of featured learning-based approaches for ICH, ICP, and MLS detection. Section 4 also discusses the state-of-the-art deep learning models for diagnosis and early management of hematoma and midline shift. Section 5 provides the discussion of CAD systems for TBI diagnosis and the various avenues for future development for automated TBI diagnosis. Finally, the conclusion of the review is presented in Section 6.

## 3. Open Source Datasets

In order to develop CAD systems to identify various pathologies associated with TBI, most of the existing studies have used smaller datasets obtained from single institutions. The two publicly available brain CT datasets that can assist in the development of machine learning algorithms to identify and categorise various brain abnormalities includes the Radiological Society of North America (RSNA) [35] and CQ500 [36]. These multi-centric and heterogeneous datasets facilitate the development of generic, automated CAD systems to assess the different types of abnormalities associated with TBI.

### 3.1. CQ500 Dataset

**Figure 4:**
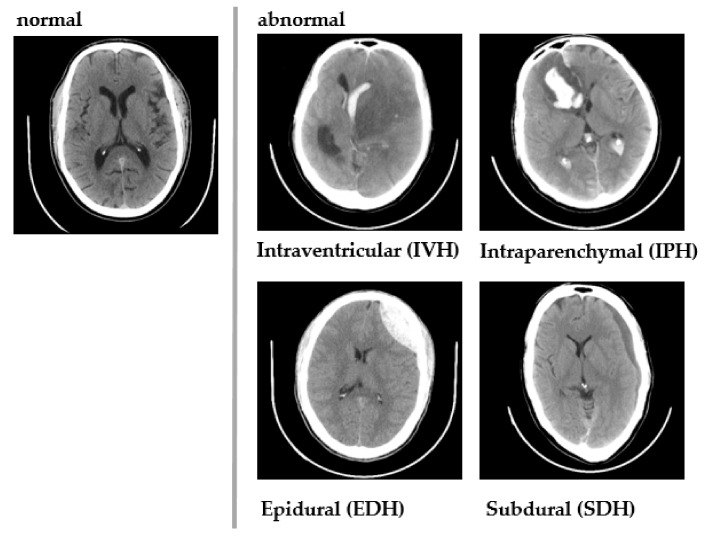
Sample CT images from CQ500 dataset.

Chilamkurthy et al. [36] created a diverse CQ500 dataset comprised of 491 brain CT scans, which were collected batch-wise from different radiology units and pooled by the Centre for Advanced Research in Images, Neurosciences and Genomics (CARING), New Delhi, India. Each CT scan was annotated by three independent radiologists for the presence or absence of (i) ICH and its five types, ICH age, and affected brain hemisphere, (ii) midline shift, and (iii) calvarial fractures. Figure 4 shows a sample of normal and abnormal images included in the dataset.

### 3.2. RSNA Dataset

The RSNA dataset is the largest publicly available dataset, consisting of 874,035 annotated brain CT images for hematoma detection and classification. Each CT image in this multi-national and multi-institutional dataset [35] is annotated by expert radiologists for the presence or absence of each of the five types of ICH. The training and test data consist of 752,803 and 121,232 CT images, respectively, with class imbalance among the subtypes of hematoma. The research studies based on these two datasets are discussed in the subsequent sections.

## 4. Generics of Computer Aided Diagnosis

Computer aided diagnosis is widely used as a part of day-to-day clinical work for the early detection and diagnosis of various abnormalities in medical imagery obtained using different imaging modalities [37]. The main focus of the CAD systems is to improve the diagnostic accuracy and consistency of radiologists in assessing the severity of TBI. The additional information about the injury that is obtained through the CAD systems will aid clinicians in making a more accurate prognosis and clinical decision. CAD systems can bring significant reduction in human error and provide quantitative and qualitative assessments of TBI in a cost-effective and rapid fashion. Thus, CAD-assisted systems facilitate the early management of TBI-related anomalies effectively and quickly, which can subsequently reduce the high rates of morbidity and mortality.

The two major approaches that are used to develop CAD systems for TBI are as follows:

Feature Learning-Based Approach

**Figure 5:**
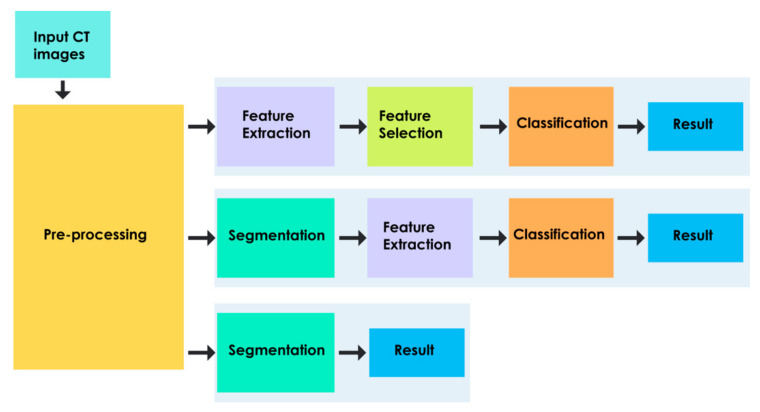
Schema of a typical feature learning-based approach for TBI.

A typical feature learning-based CAD system consists of the following stages: pre-processing, feature extraction, dimensionality reduction, and classification [38]. Pre-processing techniques can significantly improve the performance of the TBI system. Pre-processing is applied to remove noise and artefacts that are inherently present in the CT imagery, and it enhances image quality for subsequent processing [39]. Feature extraction focuses on extracting the underlying patterns of TBI in CT imagery, which is often quite challenging to detect visually. Dimensionality reduction facilitates the selection of the most pertinent features [40], which enable one to characterise the heterogeneous injuries associated with TBI. The reduced feature set is utilised by various classifiers to detect the presence of TBI-associated abnormalities and their severity. A detailed description of various stages involved in CAD systems is furnished in subsequent sections. Figure 5 shows the schema of a typical feature learning-based approach for TBI [18,23,41,42,43,44].

Deep Learning-Based Approach

**Figure 6:**
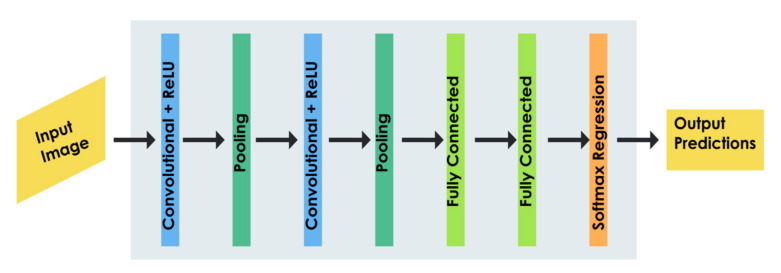
General architecture of a deep learning model for TBI diagnosis.

The convolutional neural network (CNN) has recently gained rapid attention in biomedical applications due to its self-organisation and self-learning features. As shown in Figure 6, a typical CNN or deep learning model is composed of n number of convolution layers and pooling layers, which are arranged in a successive fashion to address various applications [45]. The convolutional layers consist of convolutional filters of a fixed size to extract features from input images, and these features are accumulated and spatially reduced by a pooling layer, either using max pooling or average pooling techniques [45,46]. Thereafter, the features are propagated through the fully connected layers to the output units of the network [47,48]. The fully connected layers and the Softmax activation function are utilised for the classification of the inputs based on the reduced set of feature vectors [45,47,48].

### 4.1. Pre-Processing

Pre-processing is employed to remove the irrelevant information in the brain CT images, such as from the skull, head holder, soft tissue edema, and background, which can significantly introduce noise and degrade the performance of the CAD system. Techniques, such as intensity based thresholding, morphological operation, and connected component analysis, are used individually or combined to obtain the enhanced CT images [23,44,49,50,51]. Commonly used noise reduction techniques include median filtering and gradient magnitude filtering [23,44,50,51]. Contrast limited adaptive histogram equalisation (CLAHE) is also utilised as a technique for enhancing image quality [51]. Moreover, clustering techniques, such as K-means [52], Fuzzy c-Means (FCM) [53,54,55,56], and level-set methods, such as the distance regularised level set evolution (DRLSE) [57,58,59], are used for region of interest (ROI) extraction.

### 4.2. Feature Extraction

Various features are extracted from brain CT imagery to capture the underlying nonlinear structure. Investigators have combined the common set of features with distinct handcrafted features to improve the efficiency of the CAD system. The different methods for feature extraction are as follows:

***Texture***: Texture analysis can be done to extract and quantify the relationship among neighbouring pixels in an image. The grey level co-occurrence matrix (GLCM) [60], Gabor and Laplacian of Gaussian filters [61], and local binary patterns [62] are used for the identification of normal and abnormal images.

***Shape***: The shape of the features can play a crucial role in distinguishing the various types of TBI imagery. The major characteristics of these features include statistical independence, noise resistance, reliability, and invariance to translation, rotation, and scaling [63,64].

Discrete Wavelet Transform (DWT): The DWT is an effective mathematical tool to generate the localised time and frequency information present in CT images. The approximation and detail coefficients are generated by applying low-pass and high-pass filtering to the input signal in a successive manner, and the approximation coefficients are repeatedly used to compute wavelet features for the next scale based on the required levels of decomposition [65].

***Statistical***: Statistical features are the image properties based on the intensities of individual pixels in the CT images. As the different ranges of pixel intensity values correspond to various brain anatomical structures, these features can be used to detect the presence of abnormal components.

***Location based***: The various abnormalities associated with TBI are specific to certain brain regions, and radiologists use locational information to categorise injury. Hence, the incorporation of location-based features can aid in automated TBI diagnosis.

***Entropy based***: Entropy indicates the degree of randomness in the image pixel values [66], and a CT image with a high entropy value offers rich pixel intensity information to identify morphological differences present in the brain.

***CNN based***: Various CNN-based architectures are utilised to obtain a set of features from the TBI images. The activations of the CNN layer before the final output layer are considered as deep features for the assessment of TBI.

Texture feature extraction is widely used as a feature extraction technique, and the inclusion of shape features [67] and statistical features have proven useful for improved diagnosis of TBI [44,51,57,68,69]. Raghavendra et al. [70] extracted a set of nonlinear features based on entropy to detect the presence of intracranial hematoma in CT images and obtained an accuracy of 97.37%. Liu et al. [71] developed a classification model using wavelet, statistical, and GLCM features to detect pathological CT slices. Sharma and Venugopalan [43] have employed features based on texture, shape, and intensity to identify the subtypes of hematoma. Tong et al. [51] presented a midline formation technique to diagnose hematoma, in which LBP texture features and histogram features of both hemispheres were extracted and compared, and a recall rate of 84.86 was achieved. Rajini and Bhavani [44] proposed a model based on DWT featuring for the diagnosis of hematoma in TBI patients. Li et al. [56] concluded that the distance transform with respect to five different landmarks, along with the Bayesian classifier, can distinguish normal versus subarachnoid hematoma (SAH). Chawla et al. [69] compared image intensity features in both brain hemispheres to discriminate hematoma slices.

Another approach for detecting hematoma in CT imagery involves the segmentation of hematoma regions, and a set of usual and handcrafted features are extracted to improve the classification performance [28,34,37,72]. Shahangian et al. [42] applied a modified DRLSE to segment hematoma regions, and used handcrafted shape and texture features to classify the hematoma into four subtypes. Al-Ayoob et al. [67] developed a classification model using shape features to categorise the hematoma into three classes, and achieved an accuracy of 92%. Xiao et al. [73] proposed a different approach using primary and secondary features based on long and short axes of the largest hyperdense region to classify epidural and subdural hematoma. Yuh et al. [74] evaluated the presence of three subtypes of hematoma in segmented blood clusters based on its location, shape, and size. Zaki et al. [75] used symmetry-based location features and intensity features to classify segmented intracranial regions as bleed areas. The complete information of feature-based methods is shown in Table 3.

### 4.3. Segmentation

The hematoma segmentation in CT imagery can be realized using rule-based models [23,41,76] or machine learning models [18,27,57,58,69,77]. Chan [23] developed a knowledge-based classification system from symmetry analysis to detect acute hematoma. Ray et al. [41] combined knowledge of brain anatomy and pixel intensity distribution to segment hematoma from whole CT scan images.

The machine learning approaches can implement segmentation as a pixel/voxel-wise classification task or by combining various traditional image delineation techniques. The classification task involves extracting a set of relevant features initially to classify each pixel/voxel as hematoma, whereupon suitable post-processing techniques are then applied to obtain fine segmentation. The post-processing techniques include thresholding, morphological operations, smoothing, and active contours [18,27,57,58,69,77,78]. Farzaneh et al. [58] used handcrafted statistical, textural, and geometrical features to classify each super pixel as normal or SDH. The same research group [57] has incorporated deep features in their recent study to improve segmentation performance. Scherer et al. [68] extracted statistical and textural features to perform voxel-wise hematoma classification. Muschelli et al. [18] applied a voxel selection method based on handcrafted intensity featuring to detect ICH. Qureshi et al. [76] have tried a semi-automated approach using ANN to perform initial pixel-wise categorization with an active contour for subsequent segmentation. Yao et al. [59] generated super-pixels using the simple linear iterative clustering (SLIC) algorithm, and extracted statistical and textural features to automate hematoma segmentation. Gillebert et al. [77] performed a voxel-wise comparison of normalized CT imagery with control images, and thresholded them to obtain the lesion map.

Various hybrid segmentation approaches have been proposed to detect hematoma based on image delineation techniques, such as thresholding, region growing, FCM clustering, and active contouring [21,27,49,53,54,58,68,78]. Kumar et al. [54] explored the application of FCM clustering and entropy-based thresholding to obtain the initial hematoma image for the distance regularized level set evolution (DRLSE) method and achieved an accuracy of 99.87%. Gautam and Raman [53] evaluated the performance of a segmentation model based on white matter FCM clustering (WMFCM) and wavelet-based thresholding. Saenz et al. [50] reported a nonlinear technique that uses region growing to segment hematoma in 3D CT datasets. Bhadauria et al. [55] showed that a combination of FCM clustering and active contour modelling can lead to effective demarcation of hematoma, with an accuracy of 99.10%. Prakash et al. [27] evaluated the speed and accuracy of modified DRLSE (MDRLSE) for hematoma segmentation. Bardera et al. [78] presented a semi-automated technique that applies region growing to segment hematomas in 3D CT imagery. Zhang et al. [79] applied case-based reasoning to distinguish hematoma types occurring within the brain space, and then adaptive thresholding was used to delineate the hematoma regions. Liao et al. [80] showed that the application of several evolution rules for the modified level set method in different resolutions can be used to segment subdural hematoma (SDH).

Furthermore, Nag et al. [22] proposed a cost-effective approach that deploys an autoencoder as an unsupervised learning technique to delineate hematoma regions. The autoencoder is trained to identify the hematoma slicing for initializing the active contour Chan-Vese model, and the hematoma was segmented from the 3D CT volume with a sensitivity of 0.71. A summary of the different studies reviewed for hematoma segmentation is provided in Table 4.

### 4.4. Feature Dimensionality Reduction

Feature extraction results in a large amount of irrelevant and redundant data, which can adversely affect the performance of the automated diagnosis system [81,82]. Hence, dimensionality reduction techniques can be utilised to map the datapoints from an n-dimensional space to a lower k-dimensional space, while preserving the major characteristics. Rajini and Bhavani [44] have used principal component analysis (PCA) to select significant wavelet coefficients for more accurate and efficient classification. Shahangian et al. [42] introduced a synthetic dimensionality reduction technique by combining the Adaboost classifier and a genetic algorithm (GA). The Adaboost classifier is trained for each feature in the feature set, and the GA is utilised to determine a subset of optimal features. Raghavendra et al. [70] used Student’s *t*-test to rank the entropy-based nonlinear features and to identify significant features. Liu et al. [71] employed entropy-based feature selection to select optimal features for training the SVM classifier.

### 4.5. Classification

The aim of automated classification algorithms is to assign a class label to unknown or unseen data. The performance of a classification model can be boosted by selecting a set of powerful discriminant features that can clearly distinguish the underlying patterns of the original data.

It is evident from Table 3 and Table 4 that, in the existing studies for detection of hematoma regions, the widely used supervised classifiers are: the support vector machine (SVM) [42,51,71], random forest (RF) [18,57,69], artificial neural network (ANN) [41,76], probabilistic neural network (PNN) [70], Bayesian [56], multinomial logistic regression [67], tree bagger [58], and C4.5 [73].

In [44], various classifiers, including ANN, SVM, and k-NN, were used to detect hematoma. Shahangian et al. [42] constructed a hierarchical classifier in which the first classifier performs intraventricular hematoma (IVH) detection and the second classifier, SVM, is used for the multi-class categorisation of EDH, SDH, and ICH. In [18], multiple classification models were built to estimate the voxel-level probability of hematoma. The classifiers are logistic regression, logistic regression with penalty, a generalised additive model, and the random forest classifier. The random forest model performed well, with a median DSI value of 0.89.

### 4.6. Deep Learning for Hematoma Detection

Various machine learning methods have been applied for the detection and diagnosis of hematoma for the last two decades. It can be observed from Table 2 and Table 3 that various discriminant handcrafted features can be extracted from relatively small datasets to perform localisation and classification of hematoma. Hence, the application of these techniques for a wider, more generalised population can lead to significant error, and can result in misdiagnosis and mismanagement. Moreover, the handcrafted features are also subject to intra- and inter-observer variability, and hence, a more standardised interpretation of CT images is required for accurate and reliable prognosis and risk stratification. Moreover, some of the feature extraction techniques are complicated and computationally intensive. Recently implemented deep CNNs have shown a superior generalisation ability due to their high self-learning and self-organisation nature, without being programmed explicitly [83,84]. This subsection discusses various deep learning methods for hematoma detection in brain CT imagery.

Prevedello et al. [85] proposed a deep learning-based screening approach to identify critical test findings that includes infarct, hematoma, and hydrocephalus, in a dataset comprised of 76 images. Arbabshirani et al. [86] developed a deep learning architecture to detect hematoma in CT studies, and tested their model as a radiology workflow optimisation tool. Titano et al. [87] devised another 3D CNN model based on ResNet-50 to categorise critical and non-critical CT findings and optimise the triage workflow. Grewal et al. [88] introduced a recurrent attention DenseNet (RADnet) that incorporates slice-level context along with slice-level classification to simulate real-world hematoma detection. They have also compared the performance of a CAD system with that of human experts, resulting in an accuracy of 81.82%. Chilamkurthy et al. [36] proposed a combination of deep learning algorithms to detect, validate, and clinically test the abnormalities on non-contrast head CT using CQ500 and Qure2k datasets. A U-Net based architecture is used to localise IPH, EDH, and SDH regions, and a modified ResNet18 is applied for five-class categorisation. Dawud et al. [45] showed that a finely tuned and pre-trained AlexNet-SVM model can enhance a deep learning model for hematoma detection. Majumdar et al. [89] proposed a modified U-Net model to classify four subtypes of hematoma. Lee et al. [90] reported an ensemble model comprised of VGG16, ResNet-50, Inception-v3, and Inception-ResNet-v2 for the localisation and classification of five hematoma types. The distinct features of five-class classification include the generation of attention maps for reliable localisation and the prediction basis that justifies the model prediction. Ye et al. [91] employed an integrated approach consisting of a CNN and a recurrent neural network (RNN) to detect five subtypes of hematoma. Kuo et al. [92] proposed a patch-based, fully convolutional network (PatchFCN) that can segment and categorise hematoma with high rates of accuracy. Yao et al. [93] developed a modified U-Net based hematoma segmentation model that consists of dilated convolution. In another recent study, Yao et al. [94] applied a multi-view CNN to segment hematoma, and predicted six-month mortality using volume and shape features of segmented regions and a random forest classifier. Cho et al. [26] developed a joint approach that involves cascaded CNN to detect bleed area, and used dual FCN to categorise and segment hematoma. He [95] presented a deep learning model based on SE—ResNeXt50 and EfficientNet-B3 CNN architectures for feature extraction and five-class labelling. Ko et al. [96] developed a CNN–LSTM model for ICH identification and classification. Chang et al. [97] reported a hybrid 3D/2D mask ROI-based CNN framework with efficient hematoma detection, classification, and segmentation capabilities in parallel. Arab et al. [98] presented a deep learning model with deep supervision for quick and automated segmentation of whole-head CT. Desai et al. [99] presented a deep learning model using pre-trained Google Net to detect the presence of basal ganglia hematoma in a dataset consisting of 170 CT images. Hssayeni et al. [100] proposed a fully automated U-Net model for segmentation of hematoma regions from 82 CT scans. Irene et al. [101] developed a dynamic graph convolutional neural network model (DGCNN) to segment the bleed regions, and achieved a sensitivity of 97.8%. Anupama et al. [102] combined the GrabCut-based segmentation method and synergic deep learning to detect and classify five subtypes of hematoma. Watanabe et al. [103] developed a CAD system using U-Net to detect hematoma and reduce the reading time consumed by the physicians. Sharrock et al. [104] constructed a three-dimensional model based on VNet to segment the regions with both IVH and SDH in CT images. Mansour et al. [105] developed an automated model for ICH classification with the aid of the Inception V4 network for feature extraction and Multilayer Perceptron for five-class labelling. Kuang et al. [106] presented a semi-automated approach for segmenting both hematoma and ischemic infarct simultaneously using three different U-Net based models and multi-region contour evolution. The full details of the papers reviewed for various deep learning models for hematoma segmentation and classification are provided in Table 5.

### 4.7. Hematoma Volume Estimation

Existing clinical studies suggest that hematoma volume is a crucial factor for predicting severity and 30-day outcome [107,108]. A significant increase in hematoma volume can be observed in the first two or three hours after inception due to neurological deterioration, and the rate of growth will decrease every six hours after onset [108]. Moreover, the lesions will take various shapes over the course of time from the initial circular or ellipsoid form.

The Tada formula is one of the most widely used approaches to calculate hematoma volume in brain imagery [109]. It is given by:

(1) $$V=\frac{ABC}{2}$$

where *A* is the largest diameter of the hematoma layer, *B* is the hematoma diameter perpendicular to *A*, and *C* is the layer thickness multiplied by the number of hematoma layers. This method yields the most effective and accurate results for small and regular-shaped hematomas. The accuracy is reduced as the size increases or when lesion shapes become more irregular [110,111]. Moreover, the Tada formula has resulted in the consistent overestimation of hematoma volume, and is subject to both intra- and inter-observer variability [112,113]. Even though manual estimation of hematoma volume appears to be accurate, it is a time-consuming and arduous task, especially in the case of large clinical settings, and it may introduce inadvertent errors. Hence, automated techniques can be used as an alternative approach to facilitate rapid, accurate, and reliable quantification of ICH. This section encompasses the different techniques used for hematoma volume estimation.

Bardera et al. [78] counted the number of voxels inside the hematoma boundary and multiplied by the voxel volume to obtain the ICH volume. Scherer et al. [68] quantified the hematoma volume by summing the volume of individual voxels present in the segmented ICH. Saenz et al. [50] estimated the 3D volume of three different types of hematoma by considering the voxel size and the number of voxels present in the segmented hematoma regions. Sun and Sun [49] constructed Gengon and truncated pyramid approximation models to calculate the 3D volume of hematoma in a single patient. Farzaneh et al. [57] used the 3D resolution of the segmented ICH mask to estimate the SDH volume, resulting in underestimation of larger hematoma and overestimation of smaller ones. Chang et al. [97] developed a novel hybrid ROI-based CNN to estimate the 3D volumes of IPH, SDH, and EDH, respectively. Arab et al. [98] presented a CNN model with deep supervision (CNN–DS) to perform hematoma quantification on whole-head CT rapidly and more efficiently. Jain et al. [114] developed a U-Net based CNN model to compute the volume of acute hematoma lesions, and validated their technique using a multi-centre dataset. Irene et al. [101] combined the dynamic graph convolutional neural network model (DGCNN) and SVM with the RBF kernel to compute the hematoma volume, and achieved a mean absolute error of 99.95%. Sharrock et al. [104] used a modified VNet framework to compute the hematoma volume, and achieved a volume correlation of 0.979. Table 6 summarises the different CAD models for ICH volume quantification, namely using voxel resolution of segmented hematoma and the CNN.

### 4.8. Automated Intracranial Pressure Prediction

The current gold standard for estimating ICP involves a continuous calculation of the mean value using an invasive procedure, which can result in further complications, such as infection, meningitis, hematoma, and tissue damage [20,115]. Various non-invasive approaches have been developed in the recent years that can avoid the risks associated with invasive monitoring and the requirement of a specialised setting to measure ICP [115,116]. Existing studies related to automated non-invasive ICP (nICP) prediction and estimation can be classified into two types, namely signal-based methods and image-based methods. The signal-based methods either solely use ICP recordings or are combined with arterial blood pressure (ABP) signals, and the extracted morphological features of ICP pulses, along with other clinical data, can be employed to estimate ICP [117,118,119,120,121,122,123,124]. The inherent limitation of obtaining a large number of ICP signals to propose generalised solutions necessitates the use of widely available CT scans to evaluate ICP. The image-based methods make use of several morphological features from the CT scans to automate ICP prediction. This section presents the various machine learning-based methods applied to ICP prediction and estimation using CT scans.

Chen et al. [29] presented a texture-based approach to categorize the ICP levels of the CT scans into high versus normal using a threshold value of 15 mmHg. The proposed study applied a machine learning technique that extracts a set of 10 optimized features, and obtained a classification accuracy of 80% using SVM. Chen et al. [125] extended their study by including additional features, such as MLS, hematoma volume, and patient data, to improve the performance of two-stage ICP classification. Pappu et al. [126] designed a novel semi-automated method that computes the ratio of CSF volume to whole intracranial volume as a measure to co-relate CT features and ICP. Aghazadeh et al. [127] applied the Morlet wavelet transform to acquire textural features, and used a genetic algorithm with KNN as optimized feature selectors to label ICP as mild or severe. Qi et al. [128] developed another machine learning technique that utilized multiple features along with demographic information to categorize ICP. In another recent study by Chen et al. [129], a hybrid approach that automatically estimates MLS initially to perform ICP classification was reported. Table 7 provides a summary of the relevant CAD systems developed to analyzed ICP. It can be observed from Table 6 that deep learning neural networks have not been utilized to date to predict ICP.

### 4.9. Automated Midline Estimation

Existing studies related to midline shift can be categorized as symmetry-based methods and landmark-based methods [130]. The landmark-based methods depend on locating specific anatomical structures or landmarks to measure midline shift. These methods can iteratively seek certain landmarks within the initially identified structures. Symmetry-based methods focus on generating a deformed midline (dML), a curve that connects all the displaced midline structures of the brain, namely the SP, third ventricle, and pineal gland. Limited deep learning-based methods are reported to date to estimate MLS. The following sections discuss the various landmark-based, symmetry-based, and CNN-based methods for MLS prediction.

Yuh et al. [74] designed a suite of algorithms to evaluate CT scans of patients with suspected TBI. An MLS with greater than 5 mm is considered to be clinically significant, and is computed by evaluating the symmetry of CSF pixels with respect to the symmetry of the skull, achieving a sensitivity of 100%. Xiao et al. [80] employed a multiresolution binary level set method and expert rules to identify the regions of frontal horn, and the Hough transform was used to detect SP. Then, the distance between the most posterior location in SP and the ideal midline (iML) was considered to estimate the MLS. Chen et al. [129] developed a framework that estimates the dML based on the feature points identified in the segmented ventricles, and the horizontal shift was computed from the distance between iML and dML. Liu et al. [131] presented a technique that automatically generates a set of five optimal candidate points from the selected anatomical landmarks. All of the optimal candidate points are thus connected to form the dML, and the MLS is quantified using two measurements, namely, the area ratio and the maximum shift distance. Hosshmand et al. [28] computed the dML based on the geometrical patterns of ventricles to estimate the MLS.

Liu et al. [132] proposed a linear regression model termed H-MLS to relate the hematoma and midline shift. The hematoma is segmented from the CT images initially and using the calculated hematoma information, and the H-MLS model is used to generate the dML. Liao et al. [30] developed a procedure using a Bazier curve and genetic algorithm to automatically recognize the deformed midline (dML) in the selected CT slices at the level of foramen of Monro and measure the degree of shift. Wang et al. [133] plotted the weighted midline (WML) by assigning more weights to darker pixels in the images, and quantified the shift as the distance between the iML and the WML.

Most of the landmark-based symmetry methods failed to detect the MLS when the ICH is sufficiently large enough to destroy the symmetry of the brain, or when anatomical structures are highly deformed or missing. Very few deep learning-based methods are proposed for MLS delineation in the case of high brain deformation. Chilamkurthy et al. [36] adopted a modified ResNet18 architecture along with a random forest classifier to predict the MLS in a CT scan. Wei et al. [15] proposed a multitask learning framework that can perform skeleton extraction and regression to obtain the final midline. Nag et al. [134] developed a 2D U-Net model to segment the deformed left and right hemispheres, and various MLS indices are computed after detecting the ideal midline and the deformed midline. The complete details of CAD systems for MLS prediction/estimation is provided in Table 8.

## 5. Discussion

Traumatic brain injury is a serious neurological emergency with high rates of morbidity and mortality. A decline in patient health status begins within the first few hours after onset, and hence, delayed diagnosis highly reduces the odds of medical recovery, often leading to loss of patient life. Hence, timely diagnosis and aggressive early management of TBI is crucial. CT is the preferred modality of choice in the diagnosis of TBI due to its lower cost, high availability, and speed. The gold standard currently in practice is to manually select the required slices, delineate hematoma from the CT scanning, and quantify its volume. Even though the quantification looks accurate, it is a time-consuming and tedious task, especially in large clinical settings, and may introduce error.

**Figure 7:**
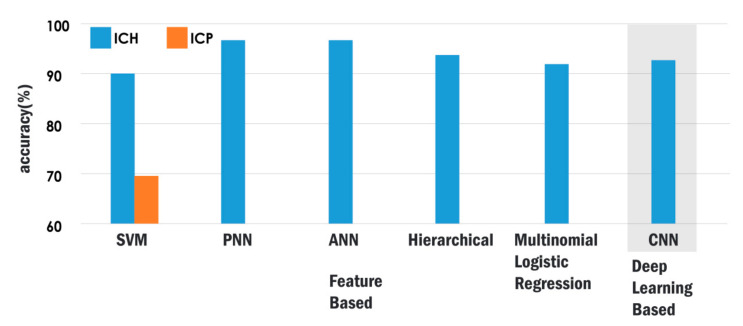
Accuracy of various automated techniques to classify ICH and ICP.

The research studies that have utilized different feature-based classification models and reported high accuracies (as outlined in Table 3) based on levels of hematoma classification and the dataset size are compared, and the best performances of these classifiers are shown in Figure 7. The deep learning models that are listed in Table 5 are also assessed based on a similar strategy, and the highest accuracy is depicted in Figure 7. It can be observed from Table 7 that the ICP classification based on SVM achieved an accuracy of 70% in a dataset consisting of 391 images, and its performance is also shown in Figure 7. As evident from the figure, the feature learning-based methods perform better in the detection and classification of hematoma with a smaller set of CT images compared to deep learning methods. No deep learning methods have been reported to date for ICP prediction.

As the hematoma starts expanding, the volume of the intracranial contents will increase, leading to raised intracranial pressure (ICP). Due to the increase in volume of intracranial contents, the brain structures will begin compressing, leading to displacement of midline structures. Hence, the MLS can be considered as a key indicator of elevated ICP.

Visual estimation of hematoma volume and midline shift by radiologists to predict ICP is a challenging task, and may lead to inaccurate and inconsistent interpretation. A CAD-assisted tool that can analyze CT images to quantify the clinical factors will surely guide the clinicians to make better decisions and an accurate prognosis. However, very few systematic studies have been reported that address the relationships between the hematoma volume, MLS, and ICP from CT imagery. Moreover, none of these methods have benchmarked their performance with radiologist-level accuracy.

### 5.1. Open Issues for Future Development

As TBI initiates a cascade of complex neurophysiological events at the cellular and sub-cellular levels, prompt diagnosis and management can considerably reduce the high rates of morbidity and mortality. Hence, various CAD schemes are developed to assist radiologists in providing quality care to the patients quickly and effectively. However, existing automated techniques to analyze the TBI-related pathologies require tremendous improvement, and some of the important issues to be addressed are as follows.

#### 5.1.1. CAD Models Based on Large and Diverse Datasets

There is a need for large, publicly available datasets that cover the diverse aspects associated with TBI-related pathologies. Diverse datasets can enable the CAD-assisted approaches to generate more accurate, generalized, and unbiased predictions. The dataset must incorporate five subtypes of hematoma of different sizes and the lesions that appear close to base of the skull. The dataset must also consider cases with multiple hematoma that will enable the study of the consequence of initial hematoma lesions of a specific type. Furthermore, CT images with cases such as deformed or missing ventricles and large hematoma, which destroys the brain symmetry, must be involved for the accurate detection of the MLS. Moreover, novel and efficient algorithms that can create a powerful and compact set of discriminative features from the heterogeneous datasets are required. Hence, fully automated algorithms that can quickly capture the subtle changes present in the diverse datasets using handcrafted and deep learning features are suggested.

#### 5.1.2. CAD Models for Detection, Classification, and Estimation of TBI-Related Pathologies

The algorithms that can detect and classify a group of TBI-associated pathologies in a single study require tremendous attention. The algorithms must focus on rapid detection and multi-class classification of these pathologies without bias.

Novel and accurate ICH detection algorithms that can handle similarities with respect to size, position, and textural content will enable clinicians towards early decision-making and improved patient outcome. The classification techniques can be extended to categorize all five types of hematoma, with the effective management of class imbalance problems. Moreover, very limited studies are done to automatically analyze CT images with multiple hematoma.

One of the major factors for TBI severity assessment and treatment planning is the 3D volume estimation of ICH, which is still an open and challenging research issue. The existing ABC/2 method has consistently overestimated the hematoma volume, and has high inter-observer variability errors. Most of the automated methods to assess hematoma volume are based on the ABC/2 method, and involve only 2D slices.

The gold standard procedure followed by clinicians to detect ICP elevation is external ventricular drain (EVD), a highly invasive and expensive procedure that can lead to further complications, including brain tissue damage and infections. Hence, a cost-effective, non-invasive procedure could reduce complications and enable clinicians to evaluate ICP beyond the regular ICU setup. Non-invasive procedures can also be used as a screening tool by the clinicians to obviate the need for invasive monitoring. The development of automated CAD systems to assess ICP from CT images requires the inclusion of key clinical indicators in the feature selection scheme to obtain highly accurate results. The use of deep learning techniques based on artificial intelligence is essential to estimate ICP with high levels of accuracy and efficiency.

CAD models that can quickly estimate the midline shift in conjunction with ICH and ICP can save the patient from lifetime disabilities and death. Existing CAD schemes for MLS estimation are based on either significant anatomical landmarks or symmetry of brain. These methods find limited application in real-time scenarios, particularly when the brain is highly deformed so as to break the symmetry, or when the anatomical structure is damaged and compressed. Hence, the application of CNN-based techniques to extract powerful features for MLS estimation, especially for large brain deformations, is suggested.

However, very few systematic studies have been reported that address the relationships between hematoma volume, MLS, and ICP from CT images. Moreover, very few studies have compared their performance to the accuracy level of radiologists to assess pathology. Furthermore, visual inspection and manual detection by radiologists is a hectic and strenuous task, which is again subject to both intra-observer and inter-observer variability. Thus, it is suggested to develop a robust, reliable, efficient, and fully automated CAD tool with radiologist-level accuracy to analyze real-time heterogeneous 3D CT volumes for the detection, classification, and estimation of TBI-related abnormalities in a single framework. This can provide in-depth information and guidance to make a more accurate clinical prognosis and streamline the triage process.

#### 5.1.3. CAD Models Based on Clinical Guidelines

The automated approaches for assessing TBI severity must be based on widely accepted clinical guidelines, like Marshall and Rotterdam classification schemes [4,135]. These schemes have grouped patients into various clinical outcome groups solely based on key clinical features present in CT head recordings. As these schemes are part of international guidelines for the management of severe TBI, the development of CAD tools for detection and estimation of key significant pathological features per these schemes may assist radiologists to perform rapid, accurate, and more standardised evaluation in emergency clinical settings. However, these classification schemes suffer from inter-observer variability errors and poor generalisation. Therefore, the development of highly standardised and optimised CAD techniques that combine qualitative and quantitative features, along with clinical data to generate a TBI risk index score, is suggested.

#### 5.1.4. Limitations of the Study

Some of the few limitations of the study include:

- Multiple research databases were searched to obtain the final set of papers for review. The searching process was limited to the set of keywords and their synonyms. Therefore, the study may have neglected some of the relevant works related to the automated detection and assessment of TBI-related abnormalities, like ICH, ICP, and MLS.
- The study consists of papers that are published in the English language. Hence, we have not considered relevant studies in other languages.
- TBI can result in different kinds of primary and secondary injuries, and hence, the paper is limited to CAD systems for the detection and assessment of hematoma, intracranial pressure, and midline shift.

## 6. Conclusions

In this paper, we have summarised state-of-the-art methods for the detection of hematoma, raised ICP, and midline shift. We have reviewed existing approaches based on their characteristics and performance measures. The feature learning approaches for the estimation of raised ICP using CT imagery performs reasonably well compared to existing automated techniques. The anatomical marker model for MLS estimation managed to handle difficult cases where previous algorithms have failed. Current deep learning frameworks for MLS estimation look promising, with an AUC of 0.96. The automated methods for hematoma detection and classification can be improved by incorporating algorithms to identify small and widespread hematoma, especially when they are close to the skull base. The inclusion of significant clinical featuring from CT imagery can optimise the performance of ICP estimation techniques. Prevailing MLS estimation methods can be optimised by automated selection of slices from 3D CT volumes.

## Figures and Tables

**Table 1:** General categorization of approaches employed by CAD systems to assess TBI.

Pathology	CAD Approaches	Techniques	TBI-Associated Abnormalities			
			ICH Detection	ICH Volume Estimation	ICP	MLS
TBI	Feature learning based	Feature based	✓	✓	✓	-
		Segmentation as pixel-wise/voxel-wise classification task	✓	-	-	-
		Segmentation based on image delineation	✓	-	-	-
		Landmark and symmetry based	-	-	-	✓
	Deep learning based	Classification	✓	✓	-	✓
		Segmentation	✓	-	-	-
		Segmentation and classification	✓	-	-	-

**Table 2:** Inclusion and exclusion criteria applied in the study.

Publication Category	Inclusion Criteria	Exclusion Criteria
Datasets used and study outcomes	Automated analysis of ICH, ICP, and MLS in humans due to TBI. CT imaging to perform automated detection and assessment of ICH, ICP, and MLS. Standard datasets for automated detection and assessment of ICH, ICP, and MLS.	Animal subjects. Treatment strategies related to ICH, ICP, and MLS. ICH, ICP, and MLS caused by conditions other than TBI.
Research design and methodology	Automated segmentation and binary/multiclass classification of ICH, ICP prediction and estimation, MLS detection and estimation, and tracing the deformed midline. Feature-based techniques or deep learning-based architectures for automated analysis and quantification of ICH, ICP, and MLS.	Statistical methods for detection for ICH, ICP, and MLS. Biochemical research pertaining to ICH, ICP, and MLS.
**Type**	Peer reviewed journals, conference proceedings, and systematic reviews	Scientific abstracts, letters to the editor, and articles without full text
**Period**	2007–2021	Before 2007
**Language**	English	Written in other languages

**Table 3:** Summary of different feature-based techniques for hematoma detection.

Authors	CT Dataset	Method	Classifier	Performance
Raghavendra et al. [70]	1603	Entropy-based nonlinear features	PNN	Acc: 97.37 Sen: 96.94 Spec: 97.83
Liu et al. [71]	11011	DWT features, statistical features, GLCM texture features	SVM	Acc: 80 Precision: 80.32 Recall: 88.22 Five-class
Sharma and Venugopalan [43]	100	Shape, intensity, and GLCM texture features	ANN	Acc: 97 Three-class
Tong et al. [51]	450	LBP texture features and histogram features	SVM	Acc: 90 Precision: 0.8486 Recall: 0.9682 Five-class
Rajini and Bhavani [44]	80	DWT features	SVM	Acc: 98 Sen: 98 Spec: 100
Li et al. [56]	129	Distance features based on landmarks	Bayesian	Sen: 100 Spec: 89.7
Chawla et al. [69]	35	Dissimilarity of intensity features in brain hemispheres	-	Acc: 90 Precision: 91 Recall: 90
Shahangian et al. [42]	627	MDRLSE + texture and shape features	Hierarchical classifier	Acc: 94.13 Four-class
Al-Ayoob et al. [67]	76	Thresholding + region growing + shape features	Multinomial Logistic Regression	Acc: 92 Precision: 92.5 Recall: 92.2 Three-class
Xiao et al. [73]	48	Multi-resolution thresholding + region growing + primary and derived features based on long and short axes	C4.5	Acc: 0.975 Three-class
Yuh et al. [74]	273	Thresholding, spatial filtering, and cluster analysis and classification based on location, size, and shape of clusters	-	Sen: 98 Spec: 59 Three-class
Zaki et al. [75]	720	FCM + multi-level thresholding + location and intensity features	-	Sen: 82.5%

**Table 4:** Summary of different techniques employed for hematoma segmentation.

Authors	CT Dataset	Method	Performance
Chan [23]	62	Top-hat transformation and symmetry detection for candidate detection + knowledge-based classification of normalised CT images	Sen: 100 Spec: 84.1
Liao et al. [80]	48	Multiresolution binary level set method + decision rules	Overlap rate: 82 Sen: 0.81
Ray et al. [41]	590	Knowledge driven thresholding + morphological operations + data fusion	Acc: 92.45 Sen: 93.95 Spec: 100
Farzaneh et al. [57]	110	SLIC + texture, spatial, and deep features + random forest + morphological operations + Gaussian smoothing	Precision: 76.12 Recall: 78.61 Dice coefficient: 75.35
Farzaneh et al. [58]	866	DRLSE + textural, statistical, and geometrical features + tree bagger classifier + multi-level thresholding	Sen: 85.02 Spec: 73.74
Scherer et al. [68]	58	First- and second-order statistics + texture and threshold features + random forest methodology + morphological operations + Gaussian smoothing	Concordance correlation coefficient = 0.98
Muschelli et al. [18]	10	Intensity-based predictors + random forest classifier + thresholding	DSI: 0.899
Qureshi et al. [76]	866	ANN and active contours	Jaccard Index: 0.8689 ± 0.042 Dice coefficient: 0.9169 ± 0.02
Yao et al. [59]	2433	SLIC + texture and statistical features + SVM + active contour model	Acc: 97 Precision: 0.59 Recall: 0.60
Gillebert et al. [77]	500	Threshold-based clustering + voxel-wise comparison of normalised and control Ct images using Crawford–Howell parametric *t*-test + thresholding	DSI: 0.89
Kumar et al. [54]	35	FCM clustering + entropy-based thresholding + DRLSE	Acc: 99.87 Sen: 87.06 Spec: 99.98
Gautam and Raman [53]	20	WMFCM clustering + wavelet-based thresholding	DSI: 0.82
Nag et al. [22]	48	Fuzzy-based intensifier + auto encoder + active contour Chan-Vese Model	Sen: 0.71 Jaccard Index: 0.55
Saenz et al. [50]	12	Hough transform + region growing	Jaccard Index: 0.9005
Bhadauria et al. [55]	100	FCM clustering + region-based active contour method	Sen: 79.48 Spec: 99.42 Dice coefficient = 0.8748
Prakash et al. [27]	200	Modified distance regularised level set evolution (MDRLSE)	Sen: 79.6 Spec: 99.9 AUC: 0.88
Bardera et al. [78]	18	Region growing	Matching ratio: 0.96
Zhang et al. [79]	10	Adaptive thresholding and case-based reasoning	Acc: 0.950 ± 0.015 Recall: 83.5

**Table 5:** Summary of different deep learning models for hematoma segmentation and classification.

Authors	CT Dataset	Method	Performance
Prevedello et al. [85]	76	AI-based deep learning approach	Sen: 90 Spec: 85 AUC: 0.91
Arbabshirani et al. [86]	46,583	DCNN	Sen: 71.5 Spec: 83.5 AUC: 0.846
Titano et al. [87]	37,236	3D-CNN	AUC: 0.88
Grewal et al. [88]	77	Recurrent Attention DenseNet (RADnet)	Acc: 81.82 Sen: 88 Precision: 81
Chilamkurthy et al. [36]	21,095 in Qure25k and 491 in CQ500	U-Net-based architecture + modified ResNet18 + random forest classifier	Sen: 92 Spec: 70 AUC: 0.87 Five-class
Dawud et al. [45]	12,635	Modified pre-trained AlexNet SVM model	Acc: 93.48 Sen: 95 Spec: 90 Four-class
Majumdar et al. [89]	134	Modified U-Net model	Sen: 81 Spec: 98
Lee et al. [90]	904	Ensemble model comprised of VGG16, ResNet-50, Inception-v3, and Inception-ResNet-v2	Sen: 78.3 Spec: 92.9 AUC: 95.9 Five-class
Ye et al. [91]	76,621	3D CNN-RNN	Sen: 80 Spec: 93.2 AUC: 0.93 Five-class
Kuo et al. [92]	4396	PatchFCN	AUC = 0.991 ± 0.006 Five-class
Yao et al. [93]	2433	Dilated CNN	Sen: 0.81 Spec: 0.96 Dice coefficient: 0.62
Yao et al. [94]	828	Multi-view CNN + volume and shape features + random forest classifier	Dice coefficient: 0.697
Cho et al. [26]	135,974	Cascaded CNN and dual fully convolutional networks (FCNs)	Sen: 97.91 Spec: 98.76 Five-class
He [95]	874,039 (RSNA dataset)	SE—ResNeXt50 and EfficientNet-B3 CNN architectures	Logarithmic Loss = 0.0548 Five-class
Ko et al. [96]	5,244,234 (RSNA dataset)	CNN-LSTM	Logarithmic Loss = 0.075 Acc: 93
Chang et al. [97]	536,266	Hybrid 3D/2D mask ROI-based CNN	Sen: 95 Spec: 97 AUC: 0.97 Four-class
Arab et al. [98]	64	CNN—DS	Precision: 0.85 Recall: 0.83 Dice coefficient: 0.84
Desai et al. [99]	170	Pre-trained augmented Google Net	AUC = 1.00
Hssayeni et al. [100]	82	U-Net	Sen: 97.28 Spec: 50.4 Dice coefficient: 0.31 Five-class
Irene et al. [101]	27	DGCNN	Sen: 97.8 Spec: 95.6
Anupama et al. [102]	82	GrabCut-based segmentation and synergic deep learning (GC- SDL)	Acc: 95.73 Sen: 94.01 Spec: 97.78 Five-class
Watanabe et al. [103]	40	U-Net	Acc: 87.5 Sen: 89.6 Spec: 81.2 Reading Time: 43 sec
Sharrock et al. [104]	500	3D VNET 128	Median Dice coefficient: 0.919
Mansour et al. [105]	82	Kapoor’s thresholding + elephant herd optimisation + Inception v4 network + multilayer perceptron	Acc: 95.06 Sen: 93.56 Spec: 97.56
Kuang et al. [106]	30	U-Net + multi-region contour evolution	Dice coefficient: 0.72

**Table 6:** Summary of different CAD models for hematoma volume estimation.

Authors	CT Dataset	Method	Performance
Farzaneh et al. [57]	110	3D resolution of the segmented ICH mask	F1: 98.22 Recall: 98.81 Spec: 92.31
Sun and Sun [49]	20	Gengon and truncated pyramid approximations	Processing time <2 s
Saenz et al. [50]	12	Voxel size multiplied by the number of voxels	-
Scherer et al. [68]	58	Summing of voxel volumes	Concordance correlation coefficient with manual estimation = 0.99
Bardera et al. [78]	18	Individual voxel volume multiplied by the number of voxels	Mean correspondence ratio = 0.74 and mean matching ratio = 0.80
Deep Learning-Based Methods			
Chang et al. [97]	536,266	Hybrid 3D/2D mask ROI-based CNN	Pearson correlation coefficients: IPH = 0.999 EDH = 0.987 SAH = 0.953
Arab et al. [98]	64	CNN—DS	Average disagreement rate = 0.08 ± 0.02
Jain et al. [114]	39	U-Net based FCN	Acc: 0.92 Sen: 0.75
Irene et al. [101]	27	DGCNN + SVM with RBF kernel	Mean square error = 3.67 × 10^4^
Sharrock et al. [104]	500	3D VNET 128	Volume correlation of 0.979 Avg. volume difference = 1.7 mL

**Table 7:** Summary of different CT-based machine learning models to evaluate ICP.

Authors	CT Dataset	Method	Performance
Chen et al. [29]	56	Texture features + SVM	Acc: 81.79 Sen: 82.25 Spec: 81.20
Chen et al. [125]	57	MLS, hematoma volume, textural patterns, and patient medical data + SVM	Acc: 70.2 Sen: 65.2 Spec: 73.7
Pappu et al. [126]	20	Segmentation of brain parenchyma + ratio of CSF to the size of intracranial vault computations (CSF_v_/ICV_v_)	Acc: 67
Aghazadeh et al. [127]	59	Fully anisotropic Morlet wavelet transform + KNN	Acc: 86.5
Qi et al. [128]	57	MLS, intracranial air cavities, ventricle size, texture patterns, blood amount, and clinical data + SVM	Acc: 73.7 Sen: 68.6 Spec: 76.6
Chen et al. [129]	391	MLS, hematoma volume, texture features, demographic information, and severity score + SVM	Acc: 70 Sen: 65 Spec: 73

**Table 8:** Summary of different CAD schemes for MLS estimation.

Authors	CT Dataset	Method	Performance
Landmark-Based Methods			
Yuh et al. [74]	273	CT density (Hounsfield units) thresholds, spatial filtering, and cluster analysis	Sen: 100 Spec: 98
Xiao et al. [80]	80	Multiresolution binary level set method and Hough transform	Maximal error: 2 mm Root mean square error: 0.57 mm
Chen et al. [129]	391	Gaussian mixture model + EM + multiple regions shape matching + texture feature extraction	Acc: 70 Sen: 65 Spec: 73
Liu et al. [102]	7040	Anatomical marker model and marker candidate selection using spatial features	Area ratio: 0.0766 Maximum distance: 4.738
Hooshmand et al. [28]	170	Ventricular geometric patterns and anatomical information	Acc: 68 Sen: 0.75 Spec: 0.65
Symmetry-Based Methods			
Liu et al. [132]	11	H-MLS	-
Liao et al. [30]	86	Bezier Curve and GA	Acc: 95
Wang et al. [133]	41	Weighted midline + maximum distance	Acc: 92.68 AUC: 0.9577
CNN-based Methods			
Chilamkurthy et al. [36]	21,095 in Qure25k and 491 in CQ500	Modified ResNet18 + random forest classifier	Sen: 0.9385 Spec: 0.907 AUC = 0.9697
Jain et al. [114]	38	U-Net based FCN	Acc: 0.89
Wei et al. [15]	640 (CQ500 and external dataset)	Regression-based line detection network (RLDN)	F1 score: 0.78 Column distance error: 1.17 Max shift distance error: 2.27
Nag et al. [134]	80	U-Net	Average error by location = 1.29 mm area = 66.4 mm^2^ volume = 253.73 mm^3^
